# Hydrogen Sulfide in Skin Diseases: A Novel Mediator and Therapeutic Target

**DOI:** 10.1155/2021/6652086

**Published:** 2021-04-20

**Authors:** Qing Xiao, Lidan Xiong, Jie Tang, Li Li, Li Li

**Affiliations:** ^1^Department of Dermatology, West China Hospital, Sichuan University, Chengdu, Sichuan 610041, China; ^2^Cosmetics Safety and Efficacy Evaluation Center, West China Hospital, Sichuan University, Chengdu, Sichuan 610041, China; ^3^Sichuan Engineering Technology Research Center of Cosmetics, Chengdu, Sichuan 610041, China; ^4^Laboratory of Pathology, West China Hospital, Sichuan University, Chengdu, Sichuan 610041, China

## Abstract

Together with nitric oxide (NO) and carbon monoxide (CO), hydrogen sulfide (H_2_S) is now recognized as a vital gaseous transmitter. The ubiquitous distributions of H_2_S-producing enzymes and potent chemical reactivities of H_2_S in biological systems make H_2_S unique in its ability to regulate cellular and organ functions in both health and disease. Acting as an antioxidant, H_2_S can combat oxidative species such as reactive oxygen species (ROS) and reactive nitrogen species (RNS) and protect the skin from oxidative stress. The aberrant metabolism of H_2_S is involved in the pathogenesis of several skin diseases, such as vascular disorders, psoriasis, ulcers, pigment disorders, and melanoma. Furthermore, H_2_S donors and some H_2_S hybrids have been evaluated in many experimental models of human disease and have shown promising therapeutic results. In this review, we discuss recent advances in understanding H_2_S and its antioxidant effects on skin pathology, the roles of altered H_2_S metabolism in skin disorders, and the potential value of H_2_S as a therapeutic intervention in skin diseases.

## 1. Introduction

The gasotransmitter hydrogen sulfide (H_2_S), recognized as the third gaseous signalling molecule along with nitric oxide (NO) and carbon monoxide (CO), is produced enzymatically in mammals under physiological conditions [1]. H_2_S was identified as a toxic gas pollutant with an odour of rotten eggs in the 18th century [2]. In 1996, Abe and Jincun reported the role of endogenous H_2_S in neuroregulation, which ushered in a new era of H_2_S and revealed its biological and pharmacological functions [3]. Later, a number of important biological effects of H_2_S were reported, including its vasorelaxation, antiapoptotic, anti-inflammatory, and antioxidative stress effects [3–6]. Emerging evidence has shown that endogenous H_2_S exhibits important functions by regulating multiple biological processes, particularly in the skin. Pathophysiological abnormalities related to altered H_2_S metabolism and function have been demonstrated in various dermatoses, such as psoriasis, vitiligo, and even melanoma [7–11]. In this review, we summarize the latest research progress on H_2_S-mediated effects, focusing on the most recent results and mechanism of the antioxidant effect of H_2_S in various skin diseases, to provide new insights into further exploration of its therapeutic targets.

## 2. Production and Metabolism of H_2_S in the Skin

### 2.1. Production of H_2_S

**Figure 1:**
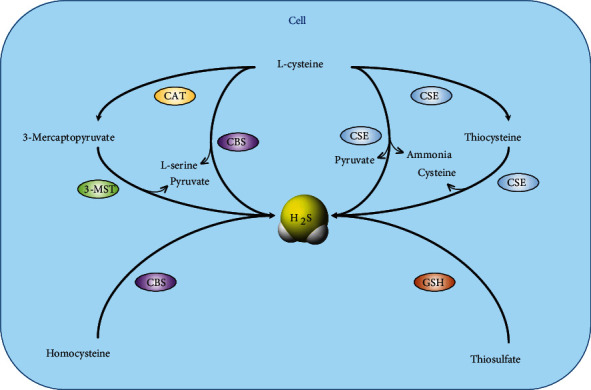
Production of H_2_S in the skin. CBS catalyses the generation of cystathionine from the substrates homocysteine and serine and liberates H_2_S from a combination of cysteine and homocysteine. CSE mainly converts cysteine into H_2_S, pyruvate, and ammonia. 3-MST generates H_2_S from 3-mercaptopyruvate produced by cysteine aminotransferase. CBS: cystathionine *β*-synthase; CSE: cystathionine *γ*-lyase; GSH: glutathione; 3-MST: 3-mercaptopyruvate sulfide transferase; CAT, cysteine aminotransferase.

H_2_S can be produced by nonenzymatic and enzymatic pathways in mammalian organisms. Nonenzymatic processes are primarily produced by the decomposition of an inorganic substance, which contributes a little to the amount of H_2_S production. The main generation of H_2_S in cutaneous tissue mostly depends on enzymatic routes using L-cysteine and homocysteine by two pyridoxal-5′-phosphate-dependent enzymes, cystathionine *β*-synthase (CBS) and cystathionine *γ*-lyase (CSE). The pyridoxal-5′-phosphate-independent enzyme, 3-mercaptopyruvate sulfurtransferase (3-MST), displays enzymatic activity at a pH of 7.4, generating H_2_S from 3-mercaptopyruvate, which is produced by cysteine aminotransferase (CAT) in mitochondria [12–14] (Figure 1).

**Figure 2:**
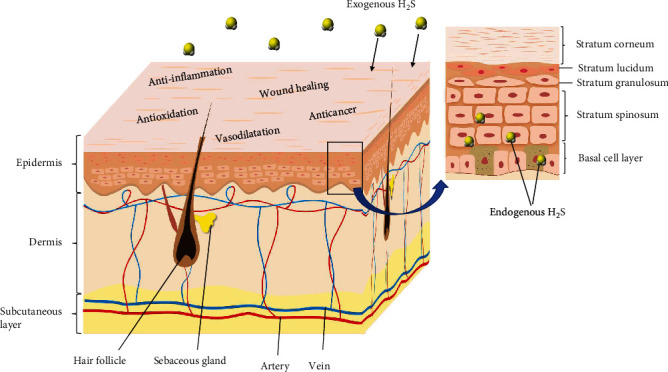
The structure of skin. The uppermost layer is the epidermis, the second layer is the dermis, and the deepest layer is the subcutaneous tissue hypodermis. The area indicated by the rectangle reveals that the epidermis is divided into five layers of cells.

The structure of the skin is shown in Figure 2. Skin has two main layers. The uppermost one is the epidermis, which is divided into five layers of cells made mostly out of keratinocytes, along with melanocytes, Merkel cells, and Langerhans cells. The dermis is the second one, a subjacent fibrous-collagenous-elastic tissue that hosts vessels, nerves, and sensory receptors. The subcutaneous tissue hypodermis is the deepest layer [15]. However, the precise localization of the H_2_S-generating enzymes in the different cell types of the skin has not yet been completely determined. Only a few reports revealed that gene expression for the H_2_S-producing enzymes occurs in normal human epidermal melanocytes and keratinocytes, which are located in the epidermis (rectangular area in Figure 2) [10, 16]. An immunohistochemical (IHC) analysis from human samples showed that CSE, CBS, and 3-MST express in normal human epidermal melanocytes, and all the dysplastic nevi were positive for CSE, negative for CBS, and variable for 3-MST [10]. CSE and 3-MST also express in the cutaneous circulation to regulate vasodilatation in humans [17, 18].

Consequently, H_2_S is currently accepted to present and exert various effects in skin, but the exact mechanism of H_2_S production in different cell types of the skin remains to be elucidated.

### 2.2. Metabolism of H_2_S

After its enzymatic synthesis, H_2_S can be either directly released or stored and liberated afterward, which maintains the proper physiological balance of its metabolism. Two forms of sulfur stores have been identified: bound sulfane sulfur and acid-labile sulfur [13, 19].

The exact details of H_2_S metabolism in the skin have not yet been elucidated. The main pathway of H_2_S catabolism is thought to occur in mitochondria by oxidation to thiosulfate and sulfate, excretion from the lung or kidney, and methylation via rhodanese and thiol methyltransferase in the cytosol [20–22]. Meanwhile, H_2_S can interact with methemoglobin to form sulfhemoglobin, a possible biomarker of plasma H_2_S [23].

## 3. The Role of H_2_S in Skin Pathophysiology

**Figure 3:**
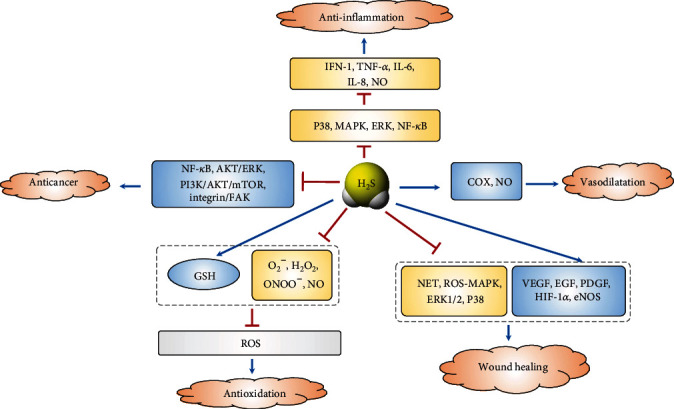
The role of H_2_S in the skin. Blue arrow: induction or stimulation; red arrow: prevention or inhibition. COX: cyclooxygenases; NO: nitric oxide; MAPK: mitogen-activated protein kinase; ERK: extracellular regulated protein kinase; NF-*κ*B: nuclear factor-*κ*B; NET: neutrophil extracellular trap; GSH: glutathione; ONOO^−^: peroxynitrite; O_2_^−^: superoxide anion; H_2_O_2_: hydrogen peroxide; ROS: reactive oxygen species; VEGF: vascular endothelial growth factor; EGF: epidermal growth factor; PDGF: platelet-derived growth factor; HIF-1*α*: hypoxia inducible factor-1*α*; eNOS: endothelial nitric oxide synthase; mTOR: mammalian target of rapamycin; Akt: protein kinase B; PI3K: phosphatidylinositol 3-kinase.

Recently, an increasing amount of evidence has illustrated the essential roles of H_2_S in vasodilatation [17], wound healing [24], inflammation [16], antioxidation [8], and the regulation of cancer cells [9, 10, 25] in the skin (Figure 3).

### 3.1. H_2_S in Vascular Disorders

In the study of vascular signalling mechanisms in healthy and sick adults, the skin is a representative and accessible regional vascular bed that modulates vascular function [26–28]. Deficits in cutaneous vascular function are highly associated with and predictive of vascular dysfunction in the coronary and renal circulation [29, 30].

CSE and 3-MST have been suggested to be expressed in the human microvasculature [17]. One recent study indicated that NaHS and Na_2_S may result in a significant dose-dependent increase in vasodilatation in the cutaneous circulation through tetraethylammonium-sensitive calcium-dependent potassium channels and functionally interact with both the COX and NO vasodilatory signalling pathways. Furthermore, as an endothelium-derived hyperpolarizing factor, H_2_S can also cause local thermal hyperaemia and reflex vasodilatation in cutaneous blood flow [17]. In the past, these functions were thought to be mediated by only endothelial NO synthase and epoxyeicosatrienoic acid-dependent mechanisms [31, 32].

Emerging evidence suggests that H_2_S is a physiologic vasodilator and regulator of blood pressure in many other organs and tissues. Rodent experiments showed that knockdown of CSE or treatment with the CSE inhibitor D,L-propargylglycine resulted in marked hypertension [33–36]. In addition, H_2_S could protect against ischaemia/reperfusion (I/R) injury in the heart, liver, kidney, brain, intestine, stomach, hind limb, lung, and retina [37]. Therefore, targeting H_2_S or its donors might become a novel preventive and therapeutic strategy for the regulation of multiple vascular pathologies.

### 3.2. H_2_S in Inflammatory Disorders

The physical anti-inflammatory role of H_2_S has attracted extensive attention. Numerous studies have shown that endogenous and exogenous H_2_S play a critical role in the resolution of inflammation [38]. Additionally, psoriasis is a common T-cell-chronic inflammatory skin disease characterised by red, thickened plaques with overlying silver-white scales. According to Alshorafa et al., the serum levels of TNF-*α*, IL-6, and IL-8 in patients with psoriasis were higher than those in a healthy control group, and the level of H_2_S was lower than that in the healthy control group. Administration of HaCaT cells with exogenous H_2_S largely inhibited the TNF-*α*-mediated upregulation of NO, IL-6, and IL-8 in a dose-dependent manner by suppressing activation of the p38, MAPK, ERK, and NF-*κ*B pathways [7].

Moreover, hypoxia of the skin is a common physiopathological characteristic of various skin diseases, such as diabetic ulcers [39, 40], pressure ulcers [41], and varicose ulcers [42], in which inflammation and oxidative stress injury are closely related. Yang et al. used cobalt chloride (CoCl_2_) as a hypoxia-mimicking agent to treat human skin keratinocytes (HaCaT cells) and demonstrated that 100-800 *μ*M NaHS administration for 30 minutes could confer a cytoprotective effect against chemical hypoxia-induced cytotoxicity and inflammation through inhibiting the reactive oxygen species- (ROS-) activated NF-*κ*B/COX-2 signalling pathway in HaCaT cells [43].

Likewise, evidence suggests that both CSE and CBS alleviate inflammation in the skin [16]. The expression of these enzymes was upregulated in human keratinocytes treated with formaldehyde at subcytotoxic concentrations, and the H_2_S produced could, in turn, inhibit an increase in proinflammatory factors (such as MMP-1, PGE2, and IL-8), which are involved in early proinflammatory processes. In addition, in mice with the cutaneous Arthus reaction, exposure to NaHS decreased the number of neutrophils recruited to skin lesions and attenuated TNF-*α* and IFN-*γ* expression in the inflammatory reaction [44].

### 3.3. H_2_S in Wound Healing

The proliferation and differentiation of the epidermis are indispensable processes in wound repair that are often dysregulated under pathologic conditions, such as those in psoriasis, epidermal cancers, atopic dermatitis (AD), and delayed wound healing [45, 46]. The proliferation and differentiation of human keratinocytes were found to be promoted by exogenous H_2_S in a dose-dependent manner by autophagy regulation [24]. Endogenous overexpression of CSE or the addition of exogenous NaHS at increasing concentrations (0-100 *μ*M) and for increasing stimulation times (0-6 d) could also increase cell proliferation in primary human epidermal melanocytes [8].

Endogenous H_2_S has been demonstrated to promote wound healing. Researchers found that CSE expression and the H_2_S content are decreased in diabetic foot ulcers and the granulation tissues of wounds [47, 48]. The wound healing process was shown to be significantly delayed in CSE^−/−^ mice compared with CSE^+/+^ mice, and blockade of CBS had the same effects, decreasing the wound closure rate and cell migration [49, 50]. In contrast, intraperitoneal treatment with NaHS and 4-hydroxythiobenzamide dramatically improved wound healing through the activation of angiopoietin-1 and restoration of endothelial progenitor cell functions in type 2 diabetic db/db mice [47]. To better understand the underlying mechanism of this effect, a recent study showed that downregulation of neutrophil extracellular trap (NET) release and blockade of ROS-induced MAPK ERK1/2 and p38 activation played an important role in the improved diabetic wound healing caused by Na_2_S [51]. Additionally, H_2_S could promote ischaemic diabetic wound healing via increasing the production of vascular endothelial growth factor (VEGF), epidermal growth factor (EGF), platelet-derived growth factor (PDGF), hypoxia inducible factor-1*α* (HIF-1*α*), and endothelial nitric oxide synthase (eNOS) in type 2 diabetic db/db mice [52]. Topical treatment with a 2% NaHS-containing ointment also accelerated wound healing by modulating angiogenesis in the granulation tissues via augmented VEGF levels in rats with streptozotocin-induced diabetes [53].

Furthermore, Zhao et al. suggested that improved wound healing by NaHS treatment in diabetic ob/ob mice was associated with reduced neutrophil and macrophage infiltration; decreased production of TNF-*α*, IL-6, and MMP-9; and increased collagen deposition in granulation tissues [48]. Thus, the identification of H_2_S as a small-molecule stimulus for cell proliferation and wound healing provides essential information for understanding epidermal repair and disorders and offers potential targets for future therapy.

### 3.4. H_2_S in Pigment Skin Disorders and Fibrotic Disease

Accumulating evidence suggests that H_2_S may confer protective effects against various types of stimuli-triggered damage in different organs, such as the skin, heart, and brain [8, 54, 55]. One of the dominant mechanisms of H_2_S protection is its antioxidant effect, which is exerted not only by increased reduced glutathione (GSH) but also the direct scavenging of superoxide anion (O_2_^−^), hydrogen peroxide (H_2_O_2_) [56], and peroxynitrite (ONOO^−^) [57] to combat oxidative stress.

A recent study showed that 10-100 *μ*M NaHS and the overexpression of CSE could promote cell proliferation and melanin synthesis by increasing the production of melanogenic enzymes (MITF, TYR, and TRP-1) in primary human epidermal melanocytes [8]. Melanocyte regeneration and melanin synthesis play important biological roles in pigment skin disorders and processes such as vitiligo, hair greying, and albinism [58, 59]. Synthesized and deposited in melanosomes, melanin can be transported to nearby keratinocytes to induce pigmentation, protecting the epidermis and underlying fibroblasts in the dermis from ultraviolet damage, which results in photoaging, oxidative stress, and even skin cancer [60, 61]. Therefore, pharmacologic regulation of H_2_S may be a novel strategy for skin disorders caused by the loss of melanocytes and dysregulation of oxidative stress.

Excessive oxidative stress is one of the dominant causes of wound healing impairment [62]. ROS arising from inflammatory cells activates various molecular signalling pathways to block angiogenesis or cytokine secretion to delay wound healing. The antioxidant and cytoprotective effects of H_2_S are also observed in the skin wound healing process [63, 64]. For example, attenuated ROS and increased VEGF expression are the main reasons for the accelerated wound healing and improved blood flow observed after pretreatment of wounds with NaHS after an operation. NaHS also promotes skin fibroblast and keratinocyte migration by alleviating ROS and increasing mitochondrial membrane potential [65]. In addition, Na_2_S could improve diabetic wound healing by downregulating NET release and suppressing ROS-induced MAPK ERK1/2 and p38 activation [51]. In a rodent experiment, the production of endogenous H_2_S, CBS, and CSE declined largely in the early stage after wounding, but intraperitoneal injection of GYY4137 (an exogenous H_2_S donor) at 50 mg·kg^−1^·day^−1^ significantly inhibited the activation of the M1 phenotype induced by mucosal wound inflammation and accelerated wound healing by downregulating the NF-*κ*B signalling pathway [66]. Taken together, these findings suggest that exogenous H_2_S supplementation and endogenous H_2_S overexpression are potential strategies to combat oxidative stress and control inflammation to promote skin wound healing.

In addition, the physical effect of H_2_S against oxidative stress and inflammation in the development of fibrosis has attracted significant attention [67]. Abnormal H_2_S metabolism is associated with the pathogenesis of fibrosis, causing damage to the structure and function of tissues and organs. Several *in vivo* and *in vitro* studies have shown that both endogenous H_2_S levels and the expression of H_2_S-related enzymes in plasma are significantly decreased in fibrotic diseases, but supplementation with exogenous H_2_S could alleviate the severity of fibrosis in different experimental animal models [68, 69]. The cytoprotective role of H_2_S in fibrosis is mainly attributed to its antioxidant, antiapoptotic, anti-inflammatory, and fibroblast-inhibitory activities [70–73]. H_2_S could restore a normal morphologic phenotype in Werner syndrome fibroblasts by attenuating oxidative damage and modulating the mTOR pathway [74]. Wang et al. demonstrated the beneficial effects of H_2_S on systemic sclerosis-associated skin and lung fibrosis. Intraperitoneal injection of NaHS markedly reduced the expression of fibrotic biomarkers such as *α*-smooth muscle actin, collagen-I, collagen-III, fibronectin, transforming growth factor-*β*1, Smad2/3 phosphorylation, and monocyte chemoattractant protein-1 in the lung in a mouse model, suggesting that H_2_S may be a potential therapy against systemic sclerosis-related organ fibrosis in the clinic [75].

### 3.5. H_2_S in Skin Cancers

H_2_S has a crucial role in multiple types of cancers, including human melanoma [10, 25, 76]. Generally, endogenous H_2_S or a relatively low concentration of exogenous H_2_S might promote or maintain cancer cell growth over a relatively short duration, while overexpression of H_2_S-generating enzymes or exposure to a relatively high level of H_2_S donor may have an anticancer effect over a relatively long duration [25].

Immunohistochemical (IHC) analysis of over 100 human subjects showed that the CSE level was increased from the transition from nevus to primary melanoma, decreased in metastatic lesions, and almost absent in lymph node metastases, and the expression of CSE and 3-MST was significantly higher than that in normal human epidermal melanocytes [10]. Overexpression of CSE led to spontaneous apoptosis in human melanoma cells by decreasing the levels of antiapoptotic proteins and suppressing the activation of NF-*κ*B. It was also reported that an exogenous H_2_S donor—DATS, an active component in garlic oil—at 100 *μ*M inhibited the proliferation of A375 human melanoma cells by downregulating the Akt and ERK pathways. Further treatment with the CSE substrate L-cysteine at 600 mg·kg^−1^ or 50 mg·kg^−1^ DATS significantly inhibited tumour growth in mice subcutaneously injected with B16-F10 cells by 67% compared with that in the control group [10]. A recent study also found that treatment of the A375 and SK-MEL-28 human melanoma cell lines with 2 mM NaHS, a donor to H_2_S gas, for 24 hours attenuated cell proliferation and migration and arrested the cell cycle but induced cell autophagy. Suppression of autophagy by ATG7 shRNA enhanced H_2_S-regulated cell apoptosis but had no synergistic effect on cell proliferation, migration, or division. Furthermore, NaHS treatment could decrease the protein expression of p-PI3K, p-Akt, and mTOR, while insulin-like growth factor-1 (IGF-1), an activator of the PI3K/AKT/mTOR pathway, could partly reverse the changes in cellular behaviour caused by NaHS, which suggested that the inhibition of human melanoma cell development by exogenous H_2_S donors might be correlated with suppression of the PI3K/AKT/mTOR pathway [9]. Moreover, 25 *μ*M DATS inhibited the growth of both human melanoma cells and basal cell carcinoma cells by upregulating cytosolic Ca^2+^ mobilization and intracellular ROS production and decreasing the activities and protein expression of matrix metalloproteinase-2 (MMP-2) and MMP-9 without a significant effect on normal keratinocyte HaCaT cell growth [77]. Specifically, the inhibitory effect of DATS on invasion and metastasis in A375 cells was found to correlate with decreased levels of several integrin subunits and focal adhesion kinase (FAK) [78]. These results suggest that the antimetastatic potential of DATS for human melanoma cells might be due to disruption of the integrin/FAK signalling pathway.

Another H_2_S-releasing naproxen derivative, naproxen-4-hydroxybenzodithioate, at 10 and 30 *μ*M was reported to induce caspase 3-mediated apoptosis and suppress human melanoma cell proliferation, migration, invasion, and colony formation *in vitro* [79].

**Figure 4:**
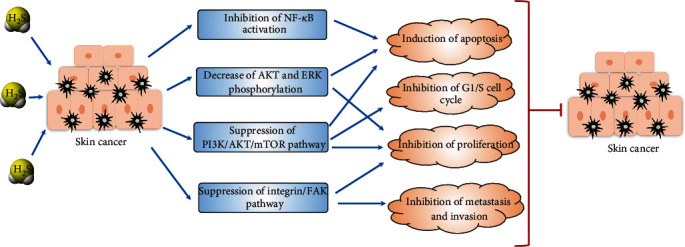
The role of H_2_S in skin cancers. The diagram shows the potential mechanisms involved in the anticancer effects. Blue arrow: induction or stimulation; red arrow: prevention or inhibition.

Nevertheless, many studies have shown that in primary melanoma, the expression of CSE is usually increased, and the overexpression of CSE in cancer cells has a tumour-promoting effect; furthermore, blockade of CSE enzymatic activity reduced proliferation rates in human melanoma cells [80]. Therefore, to achieve anticancer efficacy, further research is needed to explore a reasonable strategy for H_2_S drug application, since different types of cancer cells exhibit distinct drug susceptibilities and physiological traits.  Figure 4 shows the roles of H_2_S in skin cancers.

According to the abovementioned findings, the regulatory effects of different H_2_S donors on various skin cell types/tissues are summarized in Table 1.

## 4. Therapeutic Potential of H_2_S in Skin Diseases

In ancient times, our ancestors discovered the beneficial properties of bathing in thermal spring water, and this therapy remains a popular form of treatment for chronic skin and rheumatologic diseases today. The waters used to treat skin conditions have different physical and chemical compositions but are generally rich in sulfur, H_2_S, sulfates, and other ions [81, 82]. The activity of sulfur in the skin seems to be related mainly to its interaction with cysteine and its catabolites [83].

Bathing in sulfur-rich spring water can treat many immune-mediated skin diseases, such as psoriasis and AD. Due to the sulfur and H_2_S in thermal water, this therapy can decrease scales, pruritus, and inflammation in patients with psoriasis and exert beneficial anti-inflammatory, keratoplastic, and antipruriginous effects [84]. The use of sulfur water has also been proposed to relieve AD, leg ulcers, acne, and hidradenitis suppurativa lesions because sulfur can interact with ROS in the deeper epidermis, producing sulfur and H_2_S, which may be transformed into pentathionic acid; this pentathionic acid may be the source of the antibacterial and antifungal properties of thermal springs [85–87]. Moreover, sulfur water is therapeutic in acne patients due to its keratolytic effect, which results in peeling [88]. Therefore, bathing in sulfur-containing hot springs acts as an important alternative dermatological therapy.

Multiple recent *in vivo* and *in vitro* experiments have shed light on the biological and pharmacological roles of H_2_S under a variety of physiological and pathological conditions. An increasing number of therapeutic applications of H_2_S in skin diseases have also been revealed. As mentioned above, sulfide salts, including NaHS and Na_2_S, are the most common class of H_2_S donors applied in biological studies, providing direct, instantaneous access to the physiologically relevant forms of sulfide (H_2_S and HS^−^) [89]. Diallyl disulfide (DADS) is the active compound from allicin, and GYY4137 is a water-soluble donor used as the “gold standard” H_2_S donor in a large number of experimental studies [90]. These H_2_S donors have been or are currently under evaluation in clinical trials. A few notable examples include the naproxen-based H_2_S donors ATB-346 and naproxen-HBTA, which have been reported to exert anti-inflammatory and anticancer effects [91]. Researchers reported that 100 *μ*M ATB-346 and 30 *μ*M naproxen-HBTA could inhibit human melanoma cell proliferation, migration, invasion, and colony formation, with the possible mechanisms including inhibition of prosurvival pathways associated with NF-*κ*B and Akt activation.

Furthermore, polycaprolactone (PCL) containing jk1 was electrospun to prepare a pH-controlled H_2_S donor, generating a kind of nanofibre with a H_2_S-release function. PCL-jk1 could significantly promote wound repair and regeneration compared to that in a control group, which may have been due to the release of H_2_S, confirming the important role of H_2_S function in physiological protection of wounds [92]. In addition, Lin et al. produced an H_2_S-releasing depot formulation termed “NaHS@MPs” to treat diabetic wounds. Topical NaHS@MPs treatment of the wounds of diabetic db/db mice increased the proliferation and migration of epidermal keratinocytes, as well as angiogenesis, by inducing sustained phosphorylation of ERK1/2 and p38 and thus improved the healing of full-thickness wounds [93].

## 5. Conclusions

H_2_S, the third kind of gaseous signalling molecule, plays important roles in physiological regulatory processes in the skin (Table 1) and joins NO and CO in the group of signalling agents termed “gasotransmitters.” CSE, CBS, and 3-MST are three key H_2_S-producing enzymes that can be detected in the epidermis. Altered expression levels of these enzymes or H_2_S levels are usually associated with various skin diseases, including psoriasis, vitiligo, fibrosis, delayed wound healing, and even melanoma. Thus, CSE, CBS, and 3-MST might be biomarkers and novel molecular targets for dermatological diagnostics and treatment.

It is simultaneously becoming increasingly obvious that oxidative stress alters a number of redox-sensitive signalling pathways in different skin types, contributing to skin ageing and numerous cutaneous diseases, including skin cancers [94]. H_2_S, as an antioxidant, can protect cells from oxidative damage by decreasing the production of intracellular ROS [95]. Thus, the application of H_2_S might serve as an effective and easy method to improve these skin conditions. In addition, remarkable progress has been made in the fields of H_2_S donor chemistry and biomaterials in a short period of time since the therapeutic potential of H_2_S was discovered. Therefore, filling the knowledge gaps regarding the precise metabolic mechanism behind the role of H_2_S in skin disorders and appropriate treatment with H_2_S are key issues to be addressed, which may facilitate promising strategies for the further clinical application of H_2_S in dermatology.

## Figures and Tables

**Table 1:** Summary of the regulatory effects of different H_2_S donors on various skin cell types/tissues.

Cells/tissues	Target	H_2_S donor(s)	Effects	Ref.
Cutaneous vessels	COX and NO vasodilatory signalling pathway	NaHS and Na_2_S	Vasodilatation	[17]
HaCaT cells	p38, MAPK, ERK, and NF-*κ*B pathways	NaHS	Inhibit the TNF-*α*-mediated upregulation of NO, IL-6, and IL-8	[7]
HaCaT cells	ROS-activated NF-*κ*B/COX-2 signalling pathway	NaHS	Inhibit chemical hypoxia-induced cytotoxicity and inflammation	[43]
HaCaT cells	Autophagy	NaHS	Promote proliferation and differentiation	[25]
Endothelial progenitor cell	Angiopoietin-1	NaHS and 4-hydroxythiobenzamide	Improve diabetic wound healing	[47]
Mouse skin	NETs and ROS-induced MAPK, ERK1/2, and p38 signaling pathways	Na_2_S	Improve diabetic wound healing	[51]
Ischemic mouse adductor muscle	VEGF, EGF, PDGF, HIF-1*α*, and eNOS	Na_2_S	Promote ischemic diabetic wound healing	[52]
Granulation tissues	VEGF	NaHS-containing ointment	Accelerate diabetic wound healing	[53]
Primary human epidermal melanocytes	Melanogenic enzymes (MITF, TYR, and TRP-1)	NaHS	Promote cell proliferation and melanin synthesis	[8]
Skin fibroblasts and keratinocytes	ROS and mitochondrial membrane potential	NaHS	Accelerate wound healing and improve blood flow	[65]
Oral mucosa, peritoneal macrophages, and RAW264.7 cells	NF-*κ*B signalling pathway	GYY4137	Inhibit mucosal wound inflammation and accelerate wound healing	[66]
Skin fibroblast cell lines (AG11395 and AG12795)	mTOR pathway	NaHS	Restore a normal morphologic phenotype	[74]
Mouse skin and lung	*α*-Smooth muscle actin, collagen-I, collagen-III, fibronectin, TGF-*β*1, Smad2/3	NaHS	Alleviate the severity of systemic sclerosis-associated skin and lung fibrosis	[75]
A375 human melanoma	NF-*κ*B, AKT/ERK pathways	DATS	Inhibit melanoma proliferation, invasion, and metastasis	[10]
B16- F10 cells	AKT/ERK pathway	CSE substrate L-cysteine or DATS	Inhibit tumour growth	[10]
A375 and SK-MEL-28 human melanoma	PI3K/AKT/mTOR pathway	NaHS	Inhibit tumour growth, migration, and cell cycle	[9]
A375 and basal cell carcinoma cells	Cytosolic Ca^2+^, ROS, and mitochondrial membrane potential	DATS	Inhibit tumour growth	[77]
A375 human melanoma	Integrin/FAK pathway	DATS	Inhibit cell migration and invasion	[78]
A375 human melanoma	Caspase 3	Naproxen-HBTA	Suppress human melanoma cell proliferation, migration, invasion, and colony formation	[79]

## Data Availability

Data in this review can be found in the references part.

## Abbreviations

H_2_S: Hydrogen sulfide
NaHS: Sodium hydrosulfide
PI3K: Phosphatidylinositol 3-kinase
Akt: Protein kinase B
p-PI3K: Phosphorylated-PI3K
p-Akt: Phosphorylated-Akt
mTOR: Mammalian target of rapamycin
IGF-1: Insulin-like growth factor-1
NO: Nitric oxide
CO: Carbon monoxide
CSE: Cystathionine *γ*-lyase
CBS: Cystathionine *β*-synthase
3-MST: 3-Mercaptopyruvate sulfurtransferase
CAT: Catalase
NF-*κ*B: Nuclear factor-*κ*B
COX: Cyclooxygenase
MAPK: Mitogen-activated protein kinase
ERK: Extracellular regulated protein kinase
NET: Neutrophil extracellular trap
ROS: Reactive oxygen species
VEGF: Vascular endothelial growth factor
EGF: Epidermal growth factor
PDGF: Platelet-derived growth factor
HIF-1*α*: Hypoxia inducible factor-1*α*
eNOS: Endothelial nitric oxide synthase
MITF: Microphthalmia-associated transcription factor
TYR: Tyrosinase
TRP-1: TYR-related protein 1
TGF-*β*1: Transforming growth factor-*β*1
CoCl_2_: Cobalt chloride
DATS: Diallyl trisulfide
Naproxen-HBTA: Naproxen-4-hydroxybenzodithioate
GYY4137: Morpholin-4-ium-4-methoxyphenyl(morpholino) phosphinodithioate
GSH: Glutathione
ONOO^−^: Peroxynitrite
O_2_^−^: Superoxide anion
H_2_O_2_: Hydrogen peroxide.

## References

[1] Abe K., Kimura H. (1996). The possible role of hydrogen sulfide as an endogenous neuromodulator. *The Journal of Neuroscience*.

[2] Wang R. (2012). Physiological implications of hydrogen sulfide: a whiff exploration that blossomed. *Physiological Reviews*.

[3] Kruszyna H., Kruszyna R., Smith R. P. (1985). Cyanide and sulfide interact with nitrogenous compounds to influence the relaxation of various smooth muscles. *Proceedings of the Society for Experimental Biology and Medicine*.

[4] Xiao Q., Ying J., Xiang L., Zhang C. (2018). The biologic effect of hydrogen sulfide and its function in various diseases. *Medicine*.

[5] Zaorska E., Tomasova L., Koszelewski D., Ostaszewski R., Ufnal M. (2020). Hydrogen sulfide in pharmacotherapy, beyond the hydrogen sulfide-donors. *Biomolecules*.

[6] Wu D., Si W., Wang M., Lv S., Ji A., Li Y. (2015). Hydrogen sulfide in cancer: friend or foe?. *Nitric Oxide*.

[7] Alshorafa A. K., Guo Q., Zeng F. (2012). Psoriasis is associated with low serum levels of hydrogen sulfide, a potential anti-inflammatory molecule. *The Tohoku Journal of Experimental Medicine*.

[8] Ying J., Wang Q., Jiang M. (2020). Hydrogen sulfide promotes cell proliferation and melanin synthesis in primary human epidermal melanocytes. *Skin Pharmacology and Physiology*.

[9] Xiao Q., Ying J., Qiao Z. (2020). Exogenous hydrogen sulfide inhibits human melanoma cell development via suppression of the PI3K/AKT/ mTOR pathway. *Journal of Dermatological Science*.

[10] Panza E., de Cicco P., Armogida C. (2015). Role of the cystathionine *γ* lyase/hydrogen sulfide pathway in human melanoma progression. *Pigment Cell & Melanoma Research*.

[11] Hughes M. N., Centelles M. N., Moore K. P. (2009). Making and working with hydrogen sulfide:. *Free Radical Biology & Medicine*.

[12] Hartle M. D., Pluth M. D. (2016). A practical guide to working with H_2_S at the interface of chemistry and biology. *Chemical Society Reviews*.

[13] Singh S., Padovani D., Leslie R. A., Chiku T., Banerjee R. (2009). Relative contributions of cystathionine *β*-synthase and *γ*-cystathionase to H_2_S biogenesis via alternative trans-sulfuration reactions. *Journal of Biological Chemistry*.

[14] Yadav P. K., Yamada K., Chiku T., Koutmos M., Banerjee R. (2013). Structure and kinetic analysis of H_2_S production by human mercaptopyruvate sulfurtransferase. *The Journal of Biological Chemistry*.

[15] Arda O., Göksügür N., Tüzün Y. (2014). Basic histological structure and functions of facial skin. *Clinics in Dermatology*.

[16] Lee E., Kim H. J., Lee M. (2016). Cystathionine metabolic enzymes play a role in the inflammation resolution of human keratinocytes in response to sub-cytotoxic formaldehyde exposure. *Toxicology and Applied Pharmacology*.

[17] Kutz J. L., Greaney J. L., Santhanam L., Alexander L. M. (2015). Evidence for a functional vasodilatatory role for hydrogen sulphide in the human cutaneous microvasculature. *The Journal of Physiology*.

[18] Hosoki R., Matsuki N., Kimura H. (1997). The possible role of hydrogen sulfide as an endogenous smooth muscle relaxant in synergy with nitric oxide. *Biochemical and Biophysical Research Communications*.

[19] Ogasawara Y., Isoda S., Tanabe S. (1994). Tissue and subcellular distribution of bound and acid-labile sulfur, and the enzymic capacity for sulfide production in the rat. *Biological & Pharmaceutical Bulletin*.

[20] Stein A., Bailey S. M. (2013). Redox biology of hydrogen sulfide: implications for physiology, pathophysiology, and pharmacology. *Redox Biology*.

[21] Kabil O., Banerjee R. (2010). Redox biochemistry of hydrogen sulfide. *The Journal of Biological Chemistry*.

[22] Hildebrandt T. M., Grieshaber M. K. (2008). Three enzymatic activities catalyze the oxidation of sulfide to thiosulfate in mammalian and invertebrate mitochondria. *FEBS Journal*.

[23] Saeedi A., Najibi A., Mohammadi-Bardbori A. (2015). Effects of long-term exposure to hydrogen sulfide on human red blood cells. *The International Journal of Occupational and Environmental Medicine*.

[24] Xie X., Dai H., Zhuang B., Chai L., Xie Y., Li Y. (2016). Exogenous hydrogen sulfide promotes cell proliferation and differentiation by modulating autophagy in human keratinocytes. *Biochemical and Biophysical Research Communications*.

[25] Hellmich M. R., Szabo C. (2015). Hydrogen sulfide and cancer. *Handbook of Experimental Pharmacology*.

[26] Abularrage C. J., Sidawy A. N., Aidinian G., Singh N., Weiswasser J. M., Arora S. (2005). Evaluation of the microcirculation in vascular disease. *Journal of Vascular Surgery*.

[27] Rossi M., Carpi A., Galetta F., Franzoni F., Santoro G. (2006). The investigation of skin blood flowmotion: a new approach to study the microcirculatory impairment in vascular diseases?. *Biomedicine & Pharmacotherapy*.

[28] Holowatz L. A., Thompson-Torgerson C. S., Kenney W. L. (2008). The human cutaneous circulation as a model of generalized microvascular function. *Journal of Applied Physiology*.

[29] Khan F., Patterson D., Belch J. J., Hirata K., Lang C. C. (2008). Relationship between peripheral and coronary function using laser Doppler imaging and transthoracic echocardiography. *Clinical Science*.

[30] Coulon P., Constans J., Gosse P. (2012). Impairment of skin blood flow during post-occlusive reactive hyperhemy assessed by laser Doppler flowmetry correlates with renal resistive index. *Journal of Human Hypertension*.

[31] Bruning R. S., Santhanam L., Stanhewicz A. E. (2012). Endothelial nitric oxide synthase mediates cutaneous vasodilation during local heating and is attenuated in middle-aged human skin. *Journal of Applied Physiology*.

[32] Brunt V. E., Minson C. T. (2012). KCa channels and epoxyeicosatrienoic acids: major contributors to thermal hyperaemia in human skin. *The Journal of Physiology*.

[33] Huang P. L., Huang Z. H., Mashimo H. (1995). Hypertension in mice lacking the gene for endothelial nitric oxide synthase. *Nature*.

[34] Lake-Bruse K. D., Faraci F. M., Shesely E. G. (1999). Gene transfer of endothelial nitric oxide synthase (eNOS) in eNOS-deficient mice. *American Journal of Physiology Heart & Circulatory Physiology*.

[35] Shesel E. G. (1996). Elevated blood pressures in mice lacking endothelial nitric oxide synthase. *Proceedings of the National Academy of Sciences*.

[36] Zhao W., Ndisang J. F., Wang R., Can J. (2003). Modulation of endogenous production of H_2_S in rat tissues. *Canadian Journal of Physiology and Pharmacology*.

[37] Wu D., Wang J., Li H., Xue M., Ji A., Li Y. (2015). Role of hydrogen sulfide in ischemia-reperfusion injury. *Oxidative medicine and cellular longevity*.

[38] Wallace J. L., Ferraz J. G. P., Muscara M. N. (2012). Hydrogen sulfide: an endogenous mediator of resolution of inflammation and injury. *Antioxidants & Redox Signaling*.

[39] Barcelos L. S., Duplaa C., Kränkel N. (2009). Human CD133+ progenitor cells promote the healing of diabetic ischemic ulcers by paracrine stimulation of angiogenesis and activation of Wnt signaling. *Circulation Research*.

[40] Bolajoko E. B., Mossanda K. S., Adeniyi F., Akinosun O., Fasanmade A., Moropane M. (2008). Antioxidant and oxidative stress status in type 2 diabetes and diabetic foot ulcer. *South African Medical Journal*.

[41] Mustoe T. A., O’Shaughnessy K., Kloeters O. (2006). Chronic wound pathogenesis and current treatment strategies: a unifying hypothesis. *Plastic and Reconstructive Surgery*.

[42] Lazarides M. K., Giannoukas A. D. (2007). The role of hemodynamic measurements in the management of venous and ischemic ulcers. *The International Journal of Lower Extremity Wounds*.

[43] Yang C., Yang Z., Zhang M. (2011). Hydrogen sulfide protects against chemical hypoxia-induced cytotoxicity and inflammation in HaCaT cells through inhibition of ROS/NF-*κ*B/COX-2 pathway. *PLoS One*.

[44] Shimizu K., Ogawa F., Hara T. (2013). Exogenous application of hydrogen sulfide donor attenuates inflammatory reactions through the L-selectin-involved pathway in the cutaneous reverse passive Arthus reaction. *Journal of Leukocyte Biology s*.

[45] Angel P., Szabowski A. (2002). Function of AP-1 target genes in mesenchyma lepithelial cross-talk in skin. *Biochemical Pharmacology*.

[46] Oyoshi M. K., He R., Kumar L., Yoon J., Geha R. S. (2009). Chapter 3 Cellular and Molecular Mechanisms in Atopic Dermatitis. *Adv Immunol*.

[47] Liu F., Chen D. D., Sun X. (2014). Hydrogen sulfide improves wound healing via restoration of endothelial progenitor cell functions and activation of angiopoietin-1 in type 2 diabetes. *Diabetes*.

[48] Zhao H., Lu S., Chai J. (2017). Hydrogen sulfide improves diabetic wound healing in ob/ob mice via attenuating inflammation. *Journal of Diabetes and its Complications*.

[49] Papapetropoulos A., Pyriochou A., Altaany Z. (2009). Hydrogen sulfide is an endogenous stimulator of angiogenesis. *Proceedings of the National Academy of Sciences of the United States of America*.

[50] Saha S., Chakraborty P. K., Xiong X. (2016). Cystathionine *β*-synthase regulates endothelial function via protein S-sulfhydration. *FASEB Journal: Official Publication of the Federation of American Societies for Experimental Biology*.

[51] Yang C.-T., Chen L., Chen W. (2019). Hydrogen sulfide primes diabetic wound to close through inhibition of NETosis. *Molecular and Cellular Endocrinology*.

[52] Wang G., Li W. (2019). Hydrogen sulfide improves vessel formation of the ischemic adductor muscle and wound healing in diabetic <i>db/db</i> mice. *Iranian Journal of Basic Medical*.

[53] Wang G., Li W., Chen Q., Jiang Y., Lu X., Zhao X. (2015). Hydrogen sulfide accelerates wound healing in diabetic rats. *International Journal of Clinical and Experimental Pathology*.

[54] Venardos K. M., Perkins A., Headrick J., Kaye D. M. (2007). Myocardial ischemiareperfusion injury, antioxidant enzyme systems, and selenium: a review. *Current Medicinal Chemistry*.

[55] Kimura Y., Kimura H. (2004). Hydrogen sulfide protects neurons from oxidative stress. *Neuroscience Research*.

[56] Geng B., Chang L., Pan C. (2004). Endogenous hydrogen sulfide regulation of myocardial injury induced by isoproterenol. *Biochemical and Biophysical Research Communications*.

[57] Whiteman M., Armstrong J. S., Chu S. H. (2004). The novel neuromodulator hydrogen sulfide: an endogenous peroxynitrite ‘scavenger’?. *Journal of Neurochemistry*.

[58] Slominski A., Tobin D. J., Shibahara S., Wortsman J. (2004). Melanin pigmentation in mammalian skin and its hormonal regulation. *Physiological Reviews*.

[59] Nishimura E. K., Granter S. R., Fisher D. E. (2005). Mechanisms of hair graying: incomplete melanocyte stem cell maintenance in the niche. *Science*.

[60] White R. M., Zon L. I. (2008). Melanocytes in development, regeneration, and cancer. *Cell Stem Cell*.

[61] Yamaguchi Y., Brenner M., Hearing V. J. (2007). The regulation of skin pigmentation. *The Journal of Biological Chemistry*.

[62] Wu H., Li F., Wang S. (2018). Ceria nanocrystals decorated mesoporous silica nanoparticle based ROS-scavenging tissue adhesive for highly efficient regenerative wound healing. *Biomaterials*.

[63] Zhang G.-Y., Wu L.-C., Dai T. (2014). NADPH oxidase-2 is a key regulator of human dermal fibroblasts: a potential therapeutic strategy for the treatment of skin fibrosis. *Experimental Dermatology*.

[64] Jeon H. H., Yu Q., Lu Y. (2018). FOXO1 regulates VEGFA expression and promotes angiogenesis in healing wounds. *The Journal of Pathology*.

[65] Xu M., Hua Y., Qi Y., Meng G., Yang S. (2019). Exogenous hydrogen sulphide supplement accelerates skin wound healing via oxidative stress inhibition and vascular endothelial growth factor enhancement. *Experimental Dermatology*.

[66] Zhuang R., Guo L., du J., Wang S., Li J., Liu Y. (2018). Exogenous hydrogen sulfide inhibits oral mucosal wound-induced macrophage activation via the NF-*κ*B pathway. *Oral Diseases*.

[67] Fang L., Li H., Tang C., Geng B., Qi Y., Liu X. (2009). Hydrogen sulfide attenuates the pathogenesis of pulmonary fibrosis induced by bleomycin in rats. *Canadian Journal of Physiology and Pharmacology*.

[68] Tan G., Pan S., Li J. (2011). Hydrogen sulfide attenuates carbon tetrachloride-induced hepatotoxicity, liver cirrhosis and portal hypertension in rats. *PLoS ONE*.

[69] Song K., Wang F., Li Q. (2014). Hydrogen sulfide inhibits the renal fibrosis of obstructive nephropathy. *Kidney International*.

[70] Oury T. D., Thakker K., Menache M., Chang L. Y., Crapo J. D., Day B. J. (2001). Attenuation of bleomycin-induced pulmonary fibrosis by a catalytic antioxidant metalloporphyrin. *American Journal of Respiratory Cell and Molecular Biology*.

[71] Ming-Ju H., Yih-Shou H., Tzy-Yen C., Hui-Ling C. (2011). Hepatitis C virus E2 protein induce reactive oxygen species (ROS)-related fibrogenesis in the HSC-T6 hepatic stellate cell line. *Journal of Cellular Biochemistry*.

[72] Kim J., Seok Y. M., Jung K. J., Park K. M. (2009). Reactive oxygen species/oxidative stress contributes to progression of kidney fibrosis following transient ischemic injury in mice. *American Journal of Physiology: Renal Physiology*.

[73] Sunami R., Sugiyama H., Wang D. H. (2004). Acatalasemia sensitizes renal tubular epithelial cells to apoptosis and exacerbates renal fibrosis after unilateral ureteral obstruction. *American Journal of Physiology: Renal Physiology*.

[74] Talaei F., van Praag V. M., Henning R. H. (2013). Hydrogen sulfide restores a normal morphological phenotype in Werner syndrome fibroblasts, attenuates oxidative damage and modulates mTOR pathway. *Pharmacological Research*.

[75] Wang Z., Yin X., Gao L. (2016). The protective effect of hydrogen sulfide on systemic sclerosis associated skin and lung fibrosis in mice model. *Springerplus*.

[76] Lee Z. W., Teo X. Y., Tay E. Y. (2014). Utilizing hydrogen sulfide as a novel anti-cancer agent by targeting cancer glycolysis and pH imbalance. *British Journal of Pharmacology*.

[77] Wang H.-C., Yang J.-H., Hsieh S.-C., Sheen L.-Y. (2010). Allyl sulfides inhibit cell growth of skin cancer cells through induction of DNA damage mediated G2/M arrest and apoptosis. *Journal of Agricultural and Food Chemistry*.

[78] Wang H.-C., Chu Y.-L., Hsieh S.-C., Sheen L.-Y. (2017). Diallyl trisulfide inhibits cell migration and invasion of human melanoma a375 cells via inhibiting integrin/facal adhesion kinase pathway. *Environmental Toxicology*.

[79] Ercolano G., de Cicco P., Frecentese F. (2019). Anti-metastatic properties of naproxen-HBTA in a murine model of cutaneous melanoma. *Frontiers in Pharmacology*.

[80] Leikam C., Hufnagel A., Walz S. (2014). Cystathionase mediates senescence evasion in melanocytes and melanoma cells. *Oncogene*.

[81] Lin A. N., Reimer J. R., Carte D. M. (1988). Sulfur revisited. *Journal of the American Academy of Dermatology*.

[82] Valitutti S., Costellino F., Musiani P. (1990). Effect of sulphurus thermal water on T lymphocytes proliferative response. *Annals of Allergy*.

[83] Matz H., Orion E., Wolf R. (2003). Balneotherapy in dermatology. *Dermatologic Therapy*.

[84] Cacciapuoti S., Luciano M. A., Megna M. (2020). The role of thermal water in chronic skin diseases management: a review of the literature. *Journal of Clinical Medicine*.

[85] Inoue T., Inoue S., Kubata K. (1999). Bactericidal activity of manganese and iodide ions against Staphylococcus aureus: a possible treatment for acute atopic dermatitis. *Acta Dermato-Venereologica*.

[86] Akiyama H., Yamasaki O., Tada J., Kubota K., Arata J. (2000). Antimicrobial effects of acidic hot-spring water on Staphylococcus aureus strains isolated from atopic dermatitis patients. *Journal of Dermatological Science*.

[87] Scala E., di Caprio R., Cacciapuoti S. (2019). A new T helper 17 cytokine in hidradenitis suppurativa: antimicrobial and proinflammatory role of interleukin-26. *British Journal of Dermatology*.

[88] Gollnick H., Cunliffe W., Berson D. (2003). Management of acne: a report from a global alliance to improve outcomes in acne. *Journal of the American Academy of Dermatology*.

[89] Calderone V., Martelli A., Testai L., Citi V., Breschi M. C. (2016). Using hydrogen sulfide to design and develop drugs. *Expert Opinion on Drug Discovery*.

[90] Li L., Whiteman M., Guan Y. Y. (2008). Characterization of a novel, water-soluble hydrogen sulfide-releasing molecule (GYY4137): new insights into the biology of hydrogen sulfide. *Circulation*.

[91] de Cicco P., Panza E., Ercolano G. (2016). ATB-346, a novel hydrogen sulfide-releasing anti-inflammatory drug, induces apoptosis of human melanoma cells and inhibits melanoma development in vivo. *Pharmacological Research*.

[92] Wu J., Li Y., He C. (2016). Novel H_2_S releasing nanofibrous coating for in vivo dermal wound regeneration. *ACS Applied Materials & Interfaces*.

[93] Lin W. C., Huang C. C., Lin S. J. (2017). In situ depot comprising phase-change materials that can sustainably release a gasotransmitter H_2_S to treat diabetic wounds. *Biomaterials*.

[94] Baek J., Lee M.-G. (2016). Oxidative stress and antioxidant strategies in dermatology. *Redox Report*.

[95] Feng S., Zhao Y., Xian M., Wang Q. (2015). Biological thiols-triggered hydrogen sulfide releasing microfibers for tissue engineering applications. *Acta Biomaterialia*.

